# Arrhythmogenic Right Ventricular Cardiomyopathy: A Comprehensive Review

**DOI:** 10.3390/jcdd12020071

**Published:** 2025-02-13

**Authors:** Taha Shaikh, Darren Nguyen, Jasmine K. Dugal, Michael V. DiCaro, Brianna Yee, Nazanin Houshmand, KaChon Lei, Ali Namazi

**Affiliations:** 1 Department of Internal Medicine, University of Nevada Las Vegas, Las Vegas, NV 89154, USA; taha.shaikh@unlv.edu (T.S.); darren.nguyen@unlv.edu (D.N.); jasmine.dugal@unlv.edu (J.K.D.); brianna.yee@unlv.edu (B.Y.); 2 Department of Internal Medicine, Division of Cardiology, University of Nevada Las Vegas, Las Vegas, NV 89154, USA; nazanin.houshmand@unlv.edu (N.H.); kachon.lei@unlv.edu (K.L.); anamazi2886@gmail.com (A.N.)

**Keywords:** arrhythmogenic, heart disease, cardiovascular disease, cardiomyopathy, ARVC, VT, ventricular tachycardia, electrophysiology, congenital

## Abstract

Arrhythmogenic right ventricular cardiomyopathy (ARVC) is characterized by structural abnormalities, arrhythmias, and a spectrum of genetic and clinical manifestations. Clinically, ARVC is structurally distinguished by right ventricular dilation due to increased adiposity and fibrosis in the ventricular walls, and it manifests as cardiac arrhythmias ranging from non-sustained ventricular tachycardia to sudden cardiac death. Its prevalence has been estimated to range from 1 in every 1000 to 5000 people, with its large range being attributed to the variability in genetic penetrance from asymptomatic to significant burden. It is even suggested that the prevalence is underestimated, as the presence of genotypic mutations does not always lead to clinical manifestations that would facilitate diagnosis. Additionally, while set criteria have been in place since the 1990s, newer understanding of this condition and advancements in cardiac technology have prompted multiple revisions in the diagnostic criteria for ARVC. Novel discoveries of gene variants predisposing patients to ARVC have led to established screening techniques while providing insight into genetic counseling and management. This review aims to provide an overview of the genetics, pathophysiology, and clinical approach to ARVC. It will also focus on clinical presentation, ARVC diagnostic criteria, electrophysiological findings, including electrocardiogram characteristics, and imaging findings from cardiac MRI, 2D, and 3D echocardiogram. Current management options—including anti-arrhythmic medications, device indications, and ablation techniques—and the effectiveness of treatment will also be reviewed.

## 1. Introduction

Arrhythmogenic right ventricular cardiomyopathy (ARVC) is a rare disease characterized by fibrofatty infiltration of myocardial tissue, resulting in a predisposition to ventricular arrhythmias, heart failure, and sudden cardiac death. Although ARVC is a relatively rare condition with a prevalence of approximately 1 in 1000–5000 individuals globally, its clinical implications are profound [1]. ARVC is one of the leading causes of sudden cardiac death (SCD) in young individuals and athletes, with studies attributing 10–15% of SCD cases in these populations to ARVC, highlighting the importance of early diagnosis, risk stratification, and treatment [1].

ARVC is predominantly a genetic disease caused by mutations in genes, most commonly encoding desmosomal proteins. Mutations in non-desmosomal genes, including those affecting ion channels, calcium-handling proteins, and sarcomeric components, have also been implicated. The interplay between genetic mutations, environmental stressors such as high-intensity exercise, and the resultant mechanical and electrical instability underpins the pathophysiology of ARVC [2].

Clinically, ARVC can be challenging to diagnose due to its highly variable presentation. Some individuals remain asymptomatic, with the disease only being detected via family screening or genetic testing. Others present with palpitations, presyncope, syncope, or ventricular arrhythmias, with SCD sometimes being the first manifestation [3]. Advances in imaging modalities, such as cardiac MRI, and the development of robust diagnostic criteria, such as the 2010 Task Force Criteria and the 2020 Padua Criteria, have improved diagnostic accuracy [1].

Management of ARVC has evolved significantly over the past two decades. Treatment strategies now include lifestyle modifications, pharmaceutical intervention, catheter ablation, and implantable cardioverter-defibrillator placement [4]. Despite these advances, challenges persist in predicting disease progression and optimizing risk stratification.

It is important to note that ARVC is now also commonly referred to as arrhythmogenic cardiomyopathy (ACM) to reduce confusion, as the disease pathology may not have a predisposition to the right ventricle and can commonly affect the left ventricle or have bi-ventricular involvement as well. For this reason, much of the research on genetics and pathophysiology does not significantly differentiate between right or left ventricular disease. Clinical presentations, diagnosis, and treatment are also nearly indistinguishable for diseases involving the right, left, or both ventricles. In this article, we will continue with the classic name of ARVC and highlight specifics of right ventricular disease when possible. Regardless, the information presented will also apply to diseases involving the left ventricle, and it is reasonable to view this as a summary of ACM.

This narrative review aims to provide a concise yet comprehensive summary of the best available evidence surrounding the genetics, pathophysiology, diagnosis, and treatment of ARVC. Additionally, this narrative review discusses emerging therapies and highlights areas for future research to address the unmet needs of this complex and multifaceted disease.

## 2. Epidemiology

Due to the rarity of the disease and variability in penetrance leading to missed diagnosis, the frequency of ARVC ranges significantly in various reports. A large-scale study completed in Germany in 2004 monitored the frequency of ARVC diagnosis at their major county hospital over five years and concluded that ARVC had a prevalence of 1:1000 [5]. However, many experts believe the frequency is lower, at 1:5000 [1]. Within the same study conducted in Germany, the average age of diagnosis was noted to be 45.6 [5]. ARVC has a high prevalence in Europe, particularly Italy, and was initially thought to be an Italian disease. ARVC is now reported globally; however, there is a clear predominance in the white population [6].

There is also evidence that ARVC is more common amongst the male population. In the 2004 study performed in the German hospital mentioned above, a ratio of 1:1.286 was noted for female to male, though this difference may be more significant in the global population [5]. Though the exact cause remains unclear, many believe it is due to differences in exercise intensity. Indeed, more large prospective studies are needed to estimate the prevalence, gender differences, and age of presentation accurately for ARVC.

## 3. Genetics

ARVC is thought to be due to underlying genetic mutations often exacerbated by stress on cardiomyocytes. The 2019 Heart Rhythm Society guidelines for ARVC include 12 genes [7]. Most ARVC patients have mutations in desmosomal genes, though non-desmosomal mutations exist as well. It should also be noted that the strongest evidence exists for desmosomal gene mutations, and links to non-desmosomal proteins require further research to be substantiated.

A 2021 study conducted using the Clinical Genome Resource identified only eight genes with definitive or moderate evidence supporting their role in arrhythmogenic right ventricular cardiomyopathy (ARVC) pathogenesis. Of these, six genes—*JUP*, *PKP2*, *DSP*, *DSG2*, *DSC2*, and *TMEM43*—were classified as having definitive evidence, while *PLN* and *DES* demonstrated moderate evidence for their association with ARVC. Although several additional genes have been implicated in ARVC, their causal relationships remain uncertain due to limited supporting evidence. This uncertainty may reflect either a true lack of association or an insufficiency of current data, necessitating further investigation. Notably, *RYR2*, a gene encoding a sarcoplasmic reticulum protein, was previously considered to be associated with ARVC; however, recent findings have refuted this association [8].

A summary of important gene mutations are outlined in Table 1.

### 3.1. Desmosomal Mutations

ARVC’s genetic basis was initially discovered as a part of Naxos disease, which consists of a triad of symptoms. These include ARVC, wooly hair, and diffuse thickened palmar and pedal surfaces. Naxos disease was an autosomal recessive mutation in the JUP gene, which codes for plakoglobin, an essential part of the desmosome [9]. Shortly after the genetic discovery of Naxos disease, Carvajal syndrome was identified in South America. Carvajal disease presents with an identical triad of disease compared to Naxos disease, though it was found to be linked to a different gene variant, DSP. Presenting as another autosomal recessive mutation, DSP codes for desmoplakin [10]. Since then, three other desmosomal genes have been discovered: PKP2, DSC2, and DSG2. Nearly 60% of all ARVC patients have mutations in desmosomal genes. While most desmosomal mutations are autosomal dominant, the two initially discussed [11], which are autosomal recessive, have been linked to greater disease severity. PKP2, which codes for plakophilin 2, is the most common desmosomal mutation and is predicted to present in nearly 75% of the North American population with ARVC [2,12]. PKP2 is generally linked with the classical, primarily right ventricular form of ARVC [13]. DSC2 and DSG2 are rarer autosomal dominant mutations that code for desmocollin-2 and desmoglein-2, respectively [2].

Desmosomal mutations tend to have incomplete penetrance. It has been shown that only one-third of patients with desmosomal mutations develop symptoms that meet the most recent ARVC diagnostic criteria [14]. Generally, patients with desmosomal mutations have a younger onset than their non-desmosomal ARVC counterparts [2]. Multiple desmosomal mutations are not uncommon amongst the ARVC population, and patients with either homozygous, compound heterozygous, or double heterozygous mutations tend to have more severe phenotypes [15]. Women tend to exhibit a lower risk of gene expression, which may be due to lower exercise-related stress in the female population [16].

### 3.2. Non-Desmosomal Mutations

Multiple non-desmosomal genes have been linked to ARVC. Mutations in SNC5A are particularly relevant, as they code for the NAv1.5 sodium channel. NAv1.5 is involved in the cardiac action potential and may also point to the arrhythmogenic basis of ARVC. A cohort study of 281 ARVC patients found SNC5A mutations in roughly 2% of the sample population [17]. SNC5A mutations are primarily associated with Brugada syndrome and long QT syndrome. SNC5A mutations are believed to be inherited in an autosomal dominant pattern [2,14]. There remains limited evidence of SCN5A as a causative gene in ARVC.

In a 38-family study of ARVC families without desmosomal mutations, a mutation in the TTN gene encoding for the titin protein was noted to be implicated in 18% of mutations. Families with these mutations were found to be at higher risk of sudden cardiac death and severe disease. TTN has been shown to have classical right ventricular involvement with RV dilation commonly seen on echocardiograms [18]. As such, it has been commonly linked with dilated cardiomyopathy with unclear evidence regarding ARVC causation.

Three mutations in intercalated disks have also been associated with ARVC although there remains limited evidence. Autosomal dominant mutations in CTNNA3 and CDH2 code for alpha-T catenin 3 and N-cadherin 2, respectively [15]. CDH2 mutations were first identified in 2017 in a South African family, with further research supporting its presence in desmosome-negative populations [19]. The evidence for CTNNA3 is less substantial. In a 2013 study involving 76 patients, 2 were shown to have the CTNN3 genetic variation [20]. TJP1, tight junction protein 1, is a scaffolding protein that also localizes to the intercalated disk. There has been a suggestion that mutations in this protein may be linked to ARVC; however, current evidence is poor [2].

Mutations in genes encoding nuclear envelope proteins have also been associated with ARVC. LMNA codes for Lamin A and Lamin B, and TMEM43 codes for a nuclear envelope protein known as Luma. LMNA has been shown to be more frequent in ARVC populations, originally being found in 4 out of 108 desmosomal-negative patients in a 2012 study [21]. LMNA mutations are also associated with dilated cardiomyopathy and frequently show LV involvement in patients who meet ARVC diagnostic criteria [22,23]. Luma mutations have only been seen and researched in the Newfoundland population, first in 2008 [22]. Regardless, Luma mutations have shown definitive evidence as ARVC-associated genes.

Mutations in PLN, which codes for phospholamban, a key part of intracellular calcium regulation that interacts with the sarcoplasmic reticulum (SR), are also highly linked to ARVC and may play a key role in its pathogenesis. Interestingly, RYR2 mutations, which code for the ryanodine 2 receptors on SR, have also been linked to ARVC; however, evidence for this is less substantial [2,15]. PLN has been studied primarily in the Dutch population, and a 2012 study revealed its presence in up to 12% of the Dutch ARVC population, with 1 in 1500 being carriers [24]. It has also been reported outside of the Dutch population, albeit at a lower frequency [2].

Desmin, coded by the DES gene, is a cytoplasmic intermediate filament-linking protein that has also been shown to have genetic variation in the ARVC population. DES mutations are inherited primarily in an autosomal dominant fashion, as seen with most non-desmosomal mutations, but autosomal recessive inheritance has also been reported [25]. DES variants have been found in multiple population studies, and similar effects are reproducible in vitro [26]. Patients with DES-related ARVC have a wide variety of phenotypes given the genetic link of desmin mutations with myofibrillar myopathy, dilated cardiomyopathy, and various other desminopathies [25].

Mutations in TGFB3, a cytokine protein, have largely been considered as part of the pathophysiologic process behind ARVC, however, evidence remains limited. Over the years, TGFB3 mutations have been found in select ARVC patients/genetic families. TGFB3 is, importantly, the only secreted protein currently linked to ARVC [27].

### 3.3. Effect Modifiers

Key effect modifiers that tend to increase disease risk in ARVC include age, sex, and exercise. As mentioned earlier, females have lower levels of symptom presentation; however, the etiology of this remains unclear [2]. Given desmosomal issues in ARVC, it is understandable that age correlates with the weakening of cardiomyocytes, resulting in further propensity to develop ARVC physiology. Interestingly, exercise has been consistently associated with the worsening of disease. Aerobic exercise largely involves the RV and has been shown to accelerate the development of ARVC in predisposed individuals. In individuals already meeting ARVC criteria, exercise has been shown to increase the occurrence of arrhythmias and heart failure symptoms. As expected, the opposite also holds, and avoidance of exercise may lead to lower rates of ventricular arrhythmias and sudden cardiac death [28].

## 4. Pathophysiology

The pathophysiology of ARVC can be divided into its key features and presentation. ARVC is defined by fibrofatty replacement of the myocardium and ventricular arrhythmias, with cardiomyocyte cell death frequently seen on biopsy and autopsy.

### 4.1. Arrhythmogenesis

Arrhythmias are a hallmark and potentially fatal manifestation of ARVC, prompting extensive and prioritized research on this aspect. The two most researched mechanisms involve Connexin 43 (Cx43) and NAv1.5. Connexin 43 is found within gap junctions and is the major connexin present in the ventricular myocardium. Myocardial samples from ARVC patients consistently show abnormalities with Cx43 levels [29,30]. Within laboratory models, it has been demonstrated that desmosomal integrity is key for adequate Cx43 function. Cx43 requires proper microtubule function for localization, and these microtubules are organized by a protein known as EB1. EB1 binds to both microtubules and desmoplakin (DSP), a key component of the desmosome known to be linked to ARVC. DSP mutations cause altered EB1 interactions and decreased Cx43 levels [31]. It has not been researched, but a similar mechanism may occur in other desmosomal mutations.

NAv1.5 is located within intercalated disks and has also been shown to be reduced in myocardial samples of ARVC patients [32]. A link between these sodium channels and plakophilin 2, the most commonly mutated desmosomal component in ARVC, has been seen. In laboratory models, PKP2 knockdown mutations show decreased NAv1.5 levels due to their presence within the same protein complex. Ankyrin 3 is a trafficking protein that interacts with both plakophilin 2 and NAv1.5 and may also contribute to the relationship. Lastly, PKP2 is involved in NAv1.5 localization through EB1, similar to Cx43 and DSP, indicating another possible mechanism behind their linkage and possible cause of arrhythmias [33,34].

Further research needs to be performed on calcium regulation as a proposed mechanism of arrhythmogenesis. Links between PKP2 mutations and calcium homeostasis and known RYR2 and PLN mutations have been observed in the ARVC population [35]. In lab models, DSG-2 has also been shown to alter intracellular calcium activity further indicating its likely implication in ARVC [34].

The above mechanisms are thought to be primarily responsible for arrhythmias in early ARVC before substantial morphological changes have occurred. Following extensive fibrosis, necrosis, and adipocyte deposition, it is likely that arrhythmias and SCD in late ARVC are primarily due to these myocardial alterations in structure and function [36]. This is supported by the well-known knowledge of fibrosis and scar tissue leading to dangerous arrhythmias in patients, regardless of etiology. However, it is impossible to determine the extent to which the discussed cellular mechanisms remain involved.

### 4.2. Adipogenesis

There is no clear cellular source of adipocytes in ARVC patients. Given this lack of clarity, multiple possible mechanisms of adipogenesis have been studied in vitro in both cardiomyocytes and other desmosome-containing cells. These mechanisms are chosen for their known relation to adipogenesis, their position in the genome relative to known ARVC mutations, or due to clear cellular interactions with mutated proteins in ARVC [34]. The largest supported mechanism is the WNT-catenin-B1 pathway. WNT ligands typically bind to catenin-B1 and prevent degradation, allowing it to function adequately as a transcription factor. Catenin-B1 is normally present at the desmosome and is considered a paralogue of plakoglobin [37]. Plakoglobin competitively inhibits catenin-B1, and laboratory models, including mouse models, have shown that mutations in plakoglobin result in relocalization to the nucleus, which disrupts catenin-B1 and promotes adipogenesis [29]. Non-classical WNT signaling pathways may also be implicated with research previously conducted on the GTP-Rho-associated protein kinase (ROCK) pathway. As this pathway traditionally inhibits adipocyte differentiation in mesenchymal tissue, its role in ARVC has been a point of interest. Mouse models with decreased ROCK activity have shown pathogenesis similar to ARVC with the involvement of plakoglobin [38]. Interestingly, catenin-alpha 3 (CTTN3) mutations, which have been seen before in ARVC with limited evidence, are another proposed mechanism with WNT signaling. This protein also interacts with catenin-B1 and plakoglobin and has been shown to decrease WNT activity in epithelial cells. Although not yet seen in cardiomyocytes, WNT is further supported as a likely mechanism behind adipogenesis [34].

The HIPPO-Yap pathway is also a suggested mechanism behind the presence of adipocytes in ARVC. YAP is a transcriptional gene activator, along with TAZ, regulated by the Hippo kinase cascade and normally promotes resistance to apoptosis. Downstream effects of the Hippo cascade cause downregulation of YAP/TAZ genes. YAP interacts with catenin-B1 and suppresses activity when regulated by Hippo [39]. Alpha-catenin is again involved in studies that show that it regulates YAP in a Hippo-independent fashion [34]. YAP has also been shown to interact with plakoglobin directly in human cardiomyocyte models. The exact nature of this interaction is unclear, but it is further suggested to be involved in ARVC pathogenesis.

PPARy is another proposed mechanism, as activation is classically required for adipocyte differentiation. PPARy and canonical WNT signaling have demonstrated an inverse relationship in laboratory models. This is likely because PPARy has been shown to directly degrade catenin-B1 [40]. However, further studies must be conducted to determine the role of PPARy in ARVC conclusively.

Micro RNA (miRNA) research has also been conducted on ARVC heart models; however, evidence remains weak at the current time. A study of 24 ARVC heart models showed that out of 1078 micro RNAs, 21 had alterations in miRNA expression, with only 2 being associated with WNT and Hippo signaling [41]. Stronger supporting evidence was seen with PKP2 mutations in mouse models that showed abnormalities in miRNA that resulted in the promotion of adipogenesis [42].

### 4.3. Cardiomyocyte Mechanical Injury, Apoptosis, and Necrosis

A postmortem study on ARVC patients showed that in 30 cardiac autopsies, there was clear evidence of cardiomyocyte death, inflammation, and fibrofatty replacement resulting in atrophy [43]. Mouse models have shown that disease progression potentially starts shortly after birth [44]. Mechanical injury as a cause of eventual cell death is a clear conclusion given the known desmosomal mutations that may lead to the weakening of cell-to-cell junctions and cellular integrity. Pathological samples support this hypothesis by consistently showing abnormalities in cellular junction proteins and loss of cell membrane stability [21,39]. This has been reproduced with in vitro studies as well, with a decrease in paracellular stability seen in those with common ARVC mutations [34,45].

Cardiomyocyte apoptosis is commonly seen in ARVC patient tissue samples [46]. In vitro models have shown that abnormal desmosomal junctions may trigger apoptosis. This is further supported by mouse models with DSP mutations leading to ARVC physiology showing increased staining for apoptotic-specific factors [47]. PARP, a pro-apoptotic factor, has also been shown to be increased in mouse models with JUP mutations [45]. Necrosis in ARVC may be due to either apoptosis, mechanical cell injury, or both, and the exact process remains unclear. There has been clear evidence of necrosis in both patient samples and mouse models harboring desmosomal mutations [43].

### 4.4. Myocardial Fibrosis and Inflammation

Fibrosis is likely triggered by increased cellular stress in ARVC patients due to desmosomal fragility or other mutations causing loss of cellular junction integrity. Mutations in TGFB3 are classic mechanisms behind cardiac fibrosis, although there is no clear evidence of TGFB3 as a genetic causation in ARVC [34]. Mutation of the same region in mice models showed significant increases in TGFB3, supporting it as a possible mechanism of cardiac fibrosis [48].

TGFB1 has also been shown to be increased in ARVC patients, which may be another explanation for fibrotic development. Mouse models with JUP mutations have been shown to have increased TGFB1 signaling [49]. Similar findings have been seen in mouse models with PKP2 mutations and TMEM43 mutations, further supporting it as a major pathway for fibrosis in ARVC [48].

ARVC is known to present with “hot phases” in which patients present with chest pain, elevated troponin, and elevated inflammatory markers. A postmortem study of 36 cardiac autopsies of ARVC patients showed the presence of inflammatory cells in 39% of the total samples [50]. Similar to cardiac fibrosis, junctional instability likely triggers these “hot phases”. There is some evidence that mutated plakoglobin may also induce inflammatory cytokines such as TNF or IL-6, triggering an inflammatory cascade. Auto-antibodies have also been observed in ARVC patients with anti-DSG2 antibody titers, which show a positive correlation with disease severity [36]. Anti-heart and anti-intercalated disk antibodies have also been seen in ARVC patients [51]. These autoantibodies are likely to contribute to the inflammatory response, though additional research is needed.

## 5. Diagnosis

ARVC poses unique diagnostic challenges due to its heterogeneous clinical presentation. Diagnosing ARVC requires a multifaceted approach that integrates clinical judgment, genetic testing, electrophysiology studies, histopathology, and imaging techniques, as outlined by the 2010 Revised Task Force Criteria [52]. In this modified version of the original 1994 Task Force Criteria [53], quantitative criteria were developed based on comparison between patients with newly diagnosed ARVC and normal subject data. The multidisciplinary approach outlined in the Revised Task Force Criteria is essential in identifying ARVC and helps lead to early diagnosis. Most recently, in 2024, the European Task Force released new guidelines that expand and improve upon its preceding guidelines. Given the risks of arrhythmias and sudden cardiac death, particularly in young individuals and athletes, early and accurate diagnosis is essential.

### 5.1. Clinical Presentation

The clinical presentation of ARVC varies widely. Patients may be asymptomatic for years and may be diagnosed through family screening, or they may present with palpitations or syncope and be diagnosed in the context of a life-threatening arrhythmia. Sometimes, the first presenting symptom is sudden cardiac death (SCD). The most common symptoms and clinical findings of ARVC are outlined below.

#### 5.1.1. Asymptomatic

The asymptomatic phase is particularly common in younger individuals or family members of ARVC patients who undergo genetic screening. Close monitoring is essential, as the disease can become symptomatic over time, especially in the context of certain lifestyle factors such as high-intensity exercise. Most guidelines recommend annual or semi-annual follow-up in individuals with known ARVC. Modalities include annual EKG monitoring, Echocardiogram every 1–3 years, and cardiac magnetic resonance imaging every 3–5 years [54].

#### 5.1.2. Arrhythmias

Palpitations are frequently reported in ARVC and are typically caused by ventricular arrhythmias. Other symptomatic manifestations of arrhythmias include lightheadedness, dizziness, and syncope.

Premature ventricular contractions (PVCs) are frequently seen and can appear as isolated events or in clusters such as ventricular bigeminy, trigeminy, or quadrigeminy. PVCs are often the earliest abnormalities detected on EKG and serve as a marker for further diagnostic evaluation. Notably, high PVC burden is associated with increased risk of VT and VF in patients diagnosed with ARVC [55]. Monitoring PVC frequency can help assess disease progression and determine the effectiveness of anti-arrhythmic therapy.

**Figure 1:**
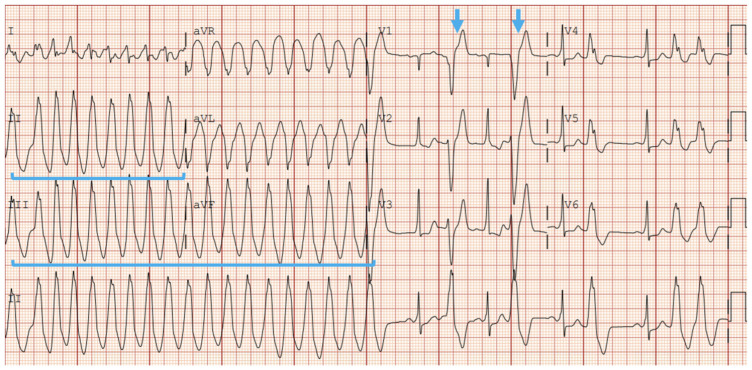
Right Ventricular Outflow Tract (RVOT) Ventricular Tachycardia, noted by Inferior Axis (positive QRS in inferior leads, blue brackets) and Left-Bundle Branch Block (blue arrow).

**Figure 2:**
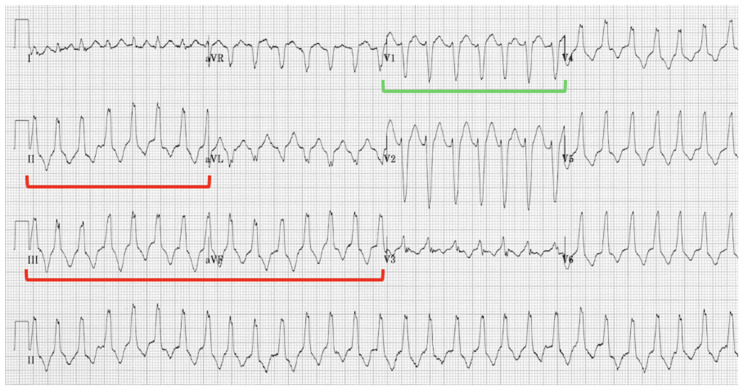
RVOT Ventricular Tachycardia with Inferior Axis (red brackets) and Left-Bundle Branch Block Morphology in V1 (green bracket).

Ventricular tachycardia (VT) can present as sustained or non-sustained ventricular tachycardia (NSVT) in patients with ARVC, and either presentation should raise suspicion of ARVC diagnosis in young, active patients. Episodes of palpitations and dizziness are frequently induced by exercise in patients with ARVC. Patients who exercised intensely showed higher rates of ventricular tachycardia, and ARVC patients who reduced their exercise showed a less frequent incidence of VT [56]. Typically, monomorphic VT is seen; however, polymorphic VT can also occur. The characteristic VT presentation on EKG in ARVC patients is typical monomorphic VT with a left bundle branch block morphology and either a non-inferior or classic right ventricular outflow tract (RVOT) pattern with an inferior axis (Figure 1 and Figure 2).

Ventricular fibrillation (VF) is more common in younger, more active patients than older patients and has also been observed more frequently in patients with multiple mutations [57]. Younger patients with documented instances of VF are, expectedly, at greater risk of recurrent VF and sudden cardiac death and should be treated with aggressive anti-arrhythmic management, including implantable cardiac defibrillator (ICD) placement [58].

#### 5.1.3. Sudden Cardiac Death

Sudden cardiac death is death originating from a cardiac source occurring at least 1 h after the onset of symptoms or a death that was unwitnessed and unexpected. ARVC is responsible for a significant percentage of SCD. Studies performed postmortem in young athletes with SCD have found ARVC to be the cause in 10–15% of cases [1]. In a case series of 66 patients diagnosed with ARVC after SCD, most cases of SCD occurred in the second decade of life. About half of these cases had a reported symptom, such as syncope, or a family history of sudden cardiac death before the SCD event. As alluded to before, exercise is recognized as a marker for poor outcomes [59]. One study estimates that about ¾ of SCD events occur during exercise [60]. As a result, guidelines recommend avoiding strenuous exercise for patients with ARVC [56]. The management of ARVC hinges on identifying and risk-stratifying high-risk patients to initiate early intervention to prevent premature death.

#### 5.1.4. Syncope

Syncope is a common symptom in patients with ARVC, occurring in 26% of patients [61]. This symptom has been recognized as an important prognostic marker for ARVC. In a case series following 92 patients with ARVC, 6 of 11 patients who had died at long-term follow-up exhibited syncopal events [62]. Furthermore, syncope was a strong predictor for ICD shock (hazard ratio 2.94, *p* = 0.013) and VT/VF (hazard ratio 3.16, *p* = 0.005) [63]. While syncope is often attributed to vasovagal causes, caution should be maintained when suspecting ARVC, and a low threshold for further evaluation of these patients is warranted, as they are at higher risk of SCD [57].

#### 5.1.5. Ventricular Dysfunction

Right ventricular or biventricular failure occurs in long-standing disease at the mean age of 39 years [3]. The phenotype depends on the patient’s location along the ARVC spectrum; patients may only have right ventricular failure or may have biventricular failure [64]. In a case series of 130 patients, 8 of whom had right ventricular failure, two exhibited concomitant left ventricular failure [65]. Progressive and refractory heart failure is another cause of death among these patients. Furthermore, more extensive ventricular involvement increases the risk of arrhythmias [60]. Apart from arrhythmias, the manifestations of RV failure can include shortness of breath, peripheral edema, or palpitations.

#### 5.1.6. Extracardiac Findings

Naxos Disease and Carvajal Disease can both present with cutaneous findings such as palmoplantar keratoderma, which is the thickening of the hands and soles and wooly hair [9]. Wooly hair is present at birth, whereas keratoderma develops over time, likely due to the pressure the infant exerts on the hands and feet [66]. These symptoms predate any cardiac manifestations.

### 5.2. Diagnostic Tools: Genetic Testing, Histopathology, EKG, Echo, cMRI, EP Study

Evaluation of this disease involves multiple tests, both laboratory and radiographic.

#### 5.2.1. EKG and Holter Monitoring

**Figure 3:**
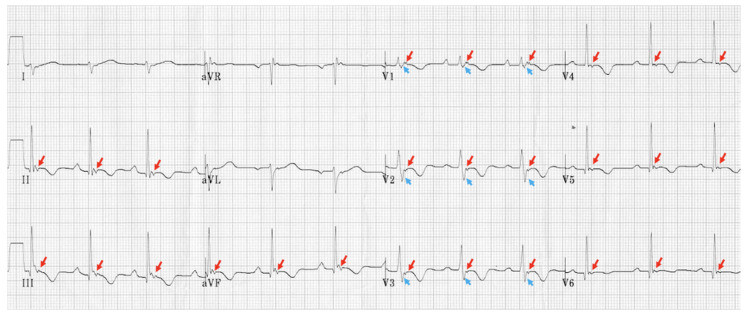
Epsilon Waves noted diffusely, most prominently in 2, 3, aVF, V1–V6 (red arrows). Prolonged S-Wave upstroke exhibited in V1, V2, V3 (blue arrows).

Specific EKG findings are classic to ARVC. Typical abnormalities seen during sinus rhythm include T-wave inversions in the V1 through V4 with the occasional extension of the inverted T-waves to V5 and V6 (which can indicate L heart involvement), and sometimes incomplete RBBB may be noted in about 15% of patients [67]. The epsilon wave, as mentioned inTable 2 and labeled in Figure 3, is commonly found in 50% of ARVC cases. During VT episodes, EKGs show an LBBB pattern with either a superior or inferior axis morphology that is not responsive to adenosine [11]. Holter long-term (>24 h) monitoring can be conducted to study arrhythmias, particularly in patients with symptoms. Holter reports often reveal intermittent ventricular arrhythmias [67]. Exercise stress testing is safe in ARVC; however, the test is believed to have low utility due to the low occurrence of sustained VT as seen in a 2010 study [68].

#### 5.2.2. Echocardiogram

A transthoracic echocardiogram can highlight structural abnormalities associated with ARVC, such as enlarged, hypokinetic, or thin right ventricular free wall, systolic akinesia, dyskinesia, or diastolic bulging in certain areas combined with RV dysfunction or RV dilatation, which are required for diagnosis, as seen in Table 2 [67]. Additionally, the degree of RVOT dilatation and the reduction of RV fractional area can be studied on echocardiography to evaluate disease severity. Typically, parameters representing RV systolic function, such as tricuspid annular plane systolic excursion (TAPSE) and peak systolic RV annular velocity, are both low in ARVC, particularly in the more advanced form of the disease [15]. Further assessment of RV function can be carried out through measurement of the global longitudinal strain (GLS) and free wall longitudinal strain (FWLS) using speckle tracking echocardiography. GLS has been shown to be highly specific for right ventricular ejection fraction. A 2015 study conducted on 60 patients showed that GLS provided the strongest correlation with cardiac MRI findings of RV function when compared to other popularly utilized measurements [69]. Additional studies suggest that GLS may be the most reproducible marker of RV function as well [70]. FWLS is also a common measurement utilized to assess RV function. A recent 2024 study suggests that FWLS may be the most specific quantitative tool for determining RV dysfunction [71]. Research is also being conducted on the potential usefulness of myocardial work (MW) in an assessment of ventricular function including RV function. Multiple studies of MW have been conducted in various populations showing the correlation between MW and the development of reduced RV function as well as other disease outcomes. Further research should be conducted in ARVC patients specifically, as well as those with other causes of isolated RV dysfunction [72].

Contrast echocardiography may improve the assessment of RV wall motion, particularly when image quality is poor. Also, 3D echocardiography and tissue deformation imaging can allow RV volume and RV ejection fraction measurements. Echocardiography should be repeated every one to three years, depending on the patient’s clinical picture. Additionally, strain-measured RV mechanical dispersion (heterogeneous contraction) may represent electrical dispersion and serve as a predictor of arrhythmic events [15].

#### 5.2.3. Cardiac Magnetic Resonance High Risk

**Figure 4:**
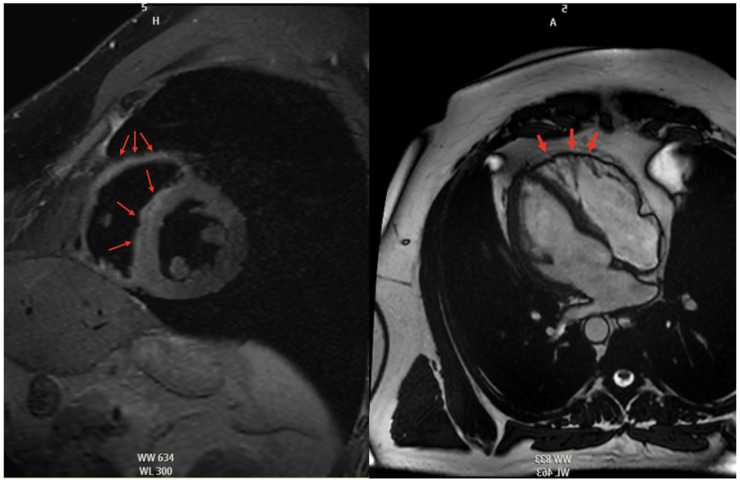
Cardiac Magnetic Resonance Images (MRI) showcasing characteristic fibrofatty infiltration of the right ventricular myocardium (red arrows). (**Left**) Fat-suppressed contrast-enhanced T1-weighted MRI. (**Right**) Steady-state free precession (SSFP) MRI.

Further imaging studies like cardiac magnetic resonance (CMR) or computed tomography (CT) in those with CMR contraindications are often utilized to visualize the right ventricle for RV volume, systolic function, and regional wall motion abnormalities [67]. Otherwise, imaging used to diagnose ARVC is based on the visual assessment of RV segmental motion anomalies and wall thickness, RV ejection fraction or fractional area, and fibrofatty replacement [14]. Fibrofatty deposition can be on CMR in Figure 4. Regardless of body size or acoustic windows, MRI can evaluate biventricular morphology, volumes, thickness, and mass, as well as regional motion, myocardial fibrous or adipose content, edema, and flow, with excellent spatial and temporal resolution. Yet, the direct visualization of adipose infiltration in the thinned RV wall has resulted in several problems as a diagnostic hallmark, given the inconsistency and poor reproducibility of these findings [14]. After regional biventricular analysis via CMR, it was found that 96% of individuals with mutation-positive ARVC had an abnormal right ventricle. The most prominent RV abnormalities found are basal inferior wall dyskinesia (94%) and basal anterior wall dyskinesia (87%) [14].

Although non-invasive imaging techniques play a pivotal role in the diagnosis and management of ARVC, the subjectivity of various imaging parameters poses major drawbacks, particularly during the assessment of wall motion abnormalities, the absence of a standardized phenotyping and reporting strategy, and sometimes the lack of imaging biomarkers tested in multiparametric models to assess the diagnosis and risk of ARVC.

#### 5.2.4. Histopathologic Findings in ARVC

Common histopathologic features of ARVC include myocyte atrophy with the deposition of both fibrous and fatty tissues in the right ventricle. Based on an analysis of postmortem transmural samples of the right ventricle, it was found that fibrous replacement of less than 60% of residual monocytes was associated with about 80% sensitivity and 95% specificity for ARVC [73]. Separate from autopsy cohort histopathologic characterization of ARVC, endomyocardial biopsy can be used to diagnose ARVC in the antemortem stage. The routine use of endomyocardial biopsy is not recommended due to its invasive nature, making its role extremely limited. This is particularly relevant in the setting of the increasing availability of extremely high-quality cardiac magnetic resonance imaging studies. Postmortem RV sampling differs from antemortem RV sampling in that a transmural specimen can be removed; however, only experienced centers may be able to perform RV free-wall biopsy, proving transmural assessment to be impractical [73].

#### 5.2.5. Role of Electrophysiologic Studies

**Figure 5:**
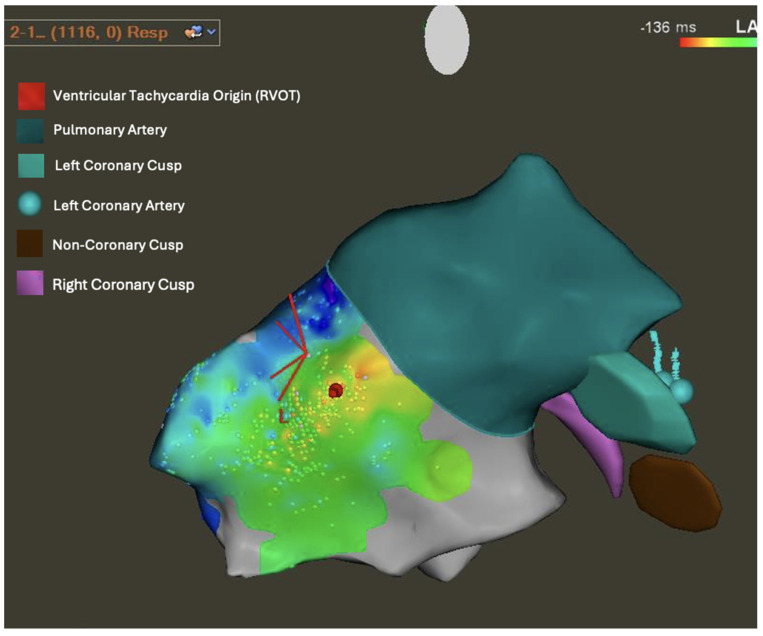
Electrophysiologic mapping revealing multifocal premature ventricular complexes arising from right ventricular outflow tract (RVOT) septum and free wall in red, and propagating outward, depicted sequentially across the color spectrum in red, orange, yellow, green, blue, and purple, with purple being the furthest from the origin point.

Electrical instability is another factor that can predict the risk of VT in ARVC. NSVT and PVC on Holter monitors are considered risk factors for sustained VT. Electrophysiological Studies (EPS), which can induce sustained VT, were shown to be a strong predictor of ICD implantation [63,74]. Furthermore, EPS reveals other conduction abnormalities associated with ARVC, such as longer ventricular activation duration and increased prevalence of PVCs [75]. These PVCs were found to originate from both ventricles, increase in frequency with exercise, and be correlated with longer repolarization and scar tissue electrical activity [75]. Examples of these aberrant conduction patterns found on EPS are depicted in Figure 5. An additional advantage of EPS is 3D electroanatomical mapping (EAM) of the right ventricle which can further increase diagnostic accuracy and guide VT ablation. Areas of low voltage noted on the 3D electroanatomic map correlate with fibrofatty replacement of the RV myocardium [76]. Additionally, EPS is more sensitive at identifying RV scarring compared to CMR [77]. Early forms of ARVC can mimic RVOT tachycardia due to the lack of RV chamber structural changes or dysfunction. EAM circumvents the limitations of functional analysis and can identify areas of RV scarring, which are pathognomonic for ARVC thereby allowing clinicians to make an early diagnosis [78].

#### 5.2.6. Multi-Modal Imaging

In 2024, the authors of the European Task Force recommended multi-modal imaging to better characterize the morpho-functional abnormalities of ARVC [79]. For example, the advantage of 2D echo centers around its ability to assess RV systolic function. This can be done through the measurement of RV global longitudinal strain and RV free wall strain, which are both reduced in ARVC [80]. Furthermore, while not included in the latest criteria, other imaging techniques such as multidetector computed tomography (MDCT), can help quantify RV volumes and systolic function, eliminating the variability of operator skill. This modality is especially useful when CMR is contraindicated or poorly tolerated by the patient. Positron emission tomography (PET) is another imaging modality that, while not included in the latest criteria, is another imaging modality to quantify RV systolic function and can serve as a risk stratification assessment tool. Patients with ARVC were found to have decreased LV uptake on iodine-123-meta-iodobenzylguanidine myocardial imaging (123-I-mIBG) and were found to have an increased risk of VT [81]. Furthermore, in a study of patients with symptomatic heart failure, those who underwent 123-I-mIBG with a heart-mediastinal uptake ratio < 1.60 had a higher risk of worsening heart failure, arrhythmias such as VT, and death [82]. Overall, while the guidelines recommend the utilization of echocardiography, angiography, and CMR as part of the diagnostic criteria, other imaging modalities should be considered as adjuncts to not only accommodate patient’s needs but also to increase diagnostic accuracy and better risk-stratify their disease, leading to better-individualized care.

#### 5.2.7. Genetic Testing in ARVC

Genetic testing is typically performed on patients who already fulfill the task force criteria for ARVC. The purpose is to obtain variant-specific cascade testing of genetic probands for all family members. Criteria for screening can vary; however, it is generally limited to first-degree relatives who have already completed clinical evaluation for ARVC [83]. Once a pathogenic or likely pathogenic gene variant is identified in an asymptomatic family member, closer surveillance testing should be warranted. It is reported that about half a percent of individuals in the general population have the pathogenic variant of ARVC.

### 5.3. Diagnostic Criteria

The original guidelines for diagnosing ARVC were published in 1994; however, due to their emphasis on right ventricular disease and excluding considerations of left ventricular disease, these guidelines were found to be highly specific with the loss of sensitivity [84]. In 2010, a revised guideline was formed to incorporate the latest research into LV manifestations of ACM and familial phenotypes into the diagnostic criteria [84]. Based on the new guidelines, a “definite” diagnosis of ARVC was made when two major criteria are fulfilled, when one major plus one minor criteria are fulfilled, or when four minor of different categories are fulfilled [52]. In a 2019 International Expert report evaluating the 2010 Task Force Criteria, the authors noted that the 2010 Criteria did not include criteria for evaluating the spectrum of ACM, suggesting to include biventricular or left-predominant disease and tissue findings suggestive of ACM with late-enhancing gadolinium enhancement on CMR [84].

Furthermore, the 2010 Task Force Criteria resulted in substantial misdiagnosis [85]. In a single-center study, 726 patients were scored utilizing the 2010 Task Force Criteria and qualified for the study if they met the definite diagnosis of ARVC. These patients were then evaluated by the center’s cardiologists for the ultimate diagnosis. Of these patients with suspected or “diagnosed” ARVC, 11.8% of these patients had their diagnosis of ARVC reversed, whereas 38.4% of these patients had ARVC ruled out when it was initially suspected [85]. As a result of their misdiagnosis of ARVC, within the 365 patients with “diagnosed” ARVC, 87 of them had ICD placed, and of this subcategory, 33 of these patients were recommended to have their ICD removed [85]. In addition, the study found that CMR misinterpretation was the most common cause of false-positive reads, especially findings of RV thinning and fat in the RV. It was noted that some conditions, such as cardiac sarcoidosis, can mimic ARVC symptoms and even fulfill the 2010 Task Force Criteria [85].

The Padua Criteria were formed in 2020 in response to the 2019 expert consensus criticisms of the 2010 Revised Task Force Criteria, which aimed to correct the flaws of the 2010 Task Force Criteria and incorporate the latest knowledge and data to better diagnose the spectrum of arrhythmogenic cardiomyopathy. This spectrum includes “dominant right”, “biventricular disease”, and “dominant left disease” [11].

One advantage of the Padua Criteria is the incorporation of new non-invasive techniques; a positive biopsy and a positive MRI are both considered major criteria in category 2 [11]. The new criteria aim to mitigate the impact of false-positive MRI readings by sorting isolated RV wall motion abnormalities into minor criteria [11]. Furthermore, there is less of a need for biopsy to diagnose ARVC, as the proposed guidelines put a positive CMR as a major criterion [11]. EKG findings such as Epsilon waves were criticized as having high observer bias, so it was changed from a major to a minor criterion [11,86]. Another important distinction the Padua Criteria provides is the emphasis on not only the number of PVCs but also the morphology of the PVC. VT originating from ARVC can have similarities to VT originating from the RVOT. One key distinction of ARVC-associated VT presents as a superior axis pattern, defined as negative QRS complexes in inferior leads (II, III, aVF) with a positive QRS complex in aVF. This superior axis finding is more specific toward ARVC and was previously listed as a major criterion in 2010 the Padua Criteria as superior axis VT can indicate the presence of RV-free wall scarring [86]. In contrast, VT inferior axis morphology, defined as positive QRS complexes in inferior leads and negative QRS in aVF, has previously been and is currently a minor criterion in the most updated Padua Criteria as it indicates idiopathic RVOT VT [87,88]. Furthermore, a PVC pattern other than LBBB of the inferior axis was associated with abnormal myocardial findings on CMR [89]. Thus, VT with LBBB of superior axis morphology is considered a major criterion in the 2020 Padua Criteria.

In 2024, the European Task Force proposed new updates to the 2020 Padua Criteria to standardize and incorporate the latest findings and data on the spectrum of ACM. Late-Gadolinium Enhancement (LGE), for example, was added as part of the major criteria for tissue characterization along with adjusting other diagnostic criteria in the other categories [79]. The authors stressed multi-modal imaging as a means to better characterize the disease and risk-stratify these patients. Furthermore, the new criteria included both RVOT VT and ARVC-related VT as part of the diagnosis, with the latter being a major criterion as it has greater specificity than the former. The changes proposed by the 2024 European Task Force compared to the 2010 International Task Force Criteria are depicted in Table 2.

## 6. Treatment: Anti-Arrhythmics, ICD Placement, Ablation, Standard of Care

### 6.1. Conservative Treatment

Restriction in physical activity remains the mainstay of conservative management among patients with ARVC, as exercise can trigger arrhythmias and accelerate disease progression, increasing the risk of SCD and heart failure. Decisions regarding the extent of exercise should involve a shared decision-making process. It is recommended that individuals limit exercise to 30 min of brisk walking per day, which corresponds to 650 MET-hours per year or less. Wang et al. performed a study that showed that exercise levels >650 MET-hours per year accelerated the phenotypic expression of ARVC [90]. Those who are genotype positive but phenotype negative should also maintain exercise restrictions to help delay the potential development of phenotypic expression [73].

### 6.2. Pharmacologic Therapy

Current pharmacological management of ARVC focuses on addressing heart failure, preventing thromboembolic events, and controlling arrhythmias. Commonly utilized medications include beta-blockers, sotalol, and guideline-directed medical therapies for heart failure, such as angiotensin-converting enzyme inhibitors (ACE inhibitors), angiotensin receptor blockers (ARBs), beta-blockers, diuretics, and anticoagulants for patients with atrial fibrillation or thromboembolic complications such as LV thrombus.

Some clinicians recommend the use of beta-blocker therapy for patients with ARVC, NSVT, or other ventricular arrhythmias as a preventative or prophylactic measure [73]. For ARVC patients with recurrent ventricular arrhythmias despite beta-blocker use, flecainide may be used as adjuvant pharmacotherapy. Other pharmacological therapies, such as amiodarone and sotalol, have been found to have conflicting results over the course of two different studies that reported the efficacy of these drugs in ARVC. Wichter et al., author of an older cohort study of 81 patients with ARVC, found that sotalol proved to be highly effective in treating inducible and non-inducible ventricular tachycardia rhythms. Amiodarone was not found to be more effective than sotalol, and given known multi-organ side effects with long-term use, it is not the drug of choice in young patients with ARVC. It is important to note that Witcher’s study of amiodarone was performed in the electrophysiology lab, which limits the ability of a full amiodarone loading dose to be administered [91].

A follow-up study on the efficacy of anti-arrhythmic drugs in ARVC from 2009 by Marcus et al. studied 95 patients receiving beta-blockers, sotalol, and amiodarone and found that neither beta-blockers nor sotalol seemed to be particularly protective against clinically relevant VT. However, patients taking amiodarone had a significantly lower risk of clinically relevant VT [92].

### 6.3. Implantable Cardioverter-Defibrillator

Implantable cardioverter-defibrillators (ICDs) are highly effective in reducing the risk of sudden cardiac death. ICD implantation is recommended for ARVC patients with (1) a history of cardiac arrest due to ventricular tachycardia (VT) or ventricular fibrillation, (2) symptomatic VT refractory to anti-arrhythmic therapy, (3) inducible VT observed during electrophysiological studies, (4) severe right and left ventricular dysfunction with poor VT tolerance, (5) non-sustained VT (NSVT) associated with syncope potentially of arrhythmic origin, or (6) a first-degree relative who experienced sudden cardiac death [67]. Although ICDs confer a 26% survival benefit, asymptomatic gene carriers without additional risk factors generally do not require prophylactic ICD placement [4].

### 6.4. Ablation

In cases of recurrent VT or frequent ICD shocks despite adequate anti-arrhythmic therapy, catheter ablation, most commonly through radiofrequency ablation, may be considered. Epicardial ablation has become widely accepted as a method of treatment in hospitals with a high volume of VT ablation treatments, as it showed a higher acute success rate compared to endocardial-only ablation [93]. Epicardial access is achieved via an anteriorly oriented subxiphoid puncture after which an ablation catheter is advanced into the pericardial space through a long J-tipped guidewire. For endocardial ablation, the pathologic substrate is found within the subtricuspid area and the RVOT. In present times, ablation energy is delivered through modern open-irrigated ablation catheters, which can achieve an ablation lesion depth of up to 8 mm. This depth should be able to create transmural lesions through the endocardial surface of the heart [94]. Although better long-term VT independence is linked to epicardial ablation, there are also more significant side effects and no discernible improvements in acute efficacy or survival [93]. However, a different 2019 study showed that long-term treatment with endocardial ablation only also provided significant symptomatic relief [94]. Regardless, the recurrence rate of VT after catheter ablation remains substantial, ranging from 50% to 75% over three years, primarily due to disease progression [95]. Most patients require several ablations over time to achieve a significant reduction of VT burden [94]. Additionally, significant exercise can alter the efficacy of ARVC treatments.

### 6.5. Cardiac Transplantation

Cardiac transplantation is reserved for patients with advanced ventricular failure unresponsive to medical therapy or recurrent, treatment-resistant arrhythmias. A recent 2023 study performed on 23 patients receiving heart transplants for ARVC showed 13 patients with end-stage biventricular dysfunction, 7 with end-stage right ventricular function only, and electrical storm in 3. The median time to transplant from diagnosis was 9 years and the median age of transplantation was 50 years old [96]. Further studies show that the number of transplants has increased over time for ARVC [97]. This could be due to increased recognition and diagnosis. Post-transplant survival rates are favorable, with 81% at five years and 77% at 10 years, comparable to patients without ARVC and exceeding outcomes for those with ischemic cardiomyopathy alone [67]. In individuals with ARVC, more information is required to determine the best time to have a heart transplant and the factors that indicate end-stage heart failure.

## 7. Future Directions

As ARVC remains a clinically rare entity, research continues to be a topic of interest. Newer advancements in technology allow for more accurate and non-invasive approaches to diagnosis, facilitating prompt management of affected individuals and their relatives. In addition to genetic testing, multi-modal imaging provides additional information to assist in diagnosis, and several imaging modalities have shown promising utility for evaluating ARVC. Although their use is not yet widespread, methods such as 3D echocardiography, multidetector computed tomography (MDCT), and nuclear imaging are proving to be useful supplements to the workup of ARVC diagnosis and may be officially incorporated into diagnostic criteria in the future [98].

As current therapies are focused on symptomatic treatment, additional research is needed to develop targeted therapies to prevent progression, avoid invasive procedures such as ablation or cardiac transplantation, and improve morbidity and mortality. With the exploration of several pathophysiological pathways, as outlined above, newer discoveries focused on the pathogenesis of ARVC will pave the way for directed therapies in the future in the hopes of extending the lifespan and improving the quality of life in affected individuals.

## 8. Conclusions

ARVC is a rare disease occurring in around 1:5000 people. Though rare, its cardiac manifestations can lead to devastating effects and are a significant cause of SCD. Despite traditionally being thought of as a European or Western disease, reports of ARVC are now being seen globally [14]. The pathology of ARVC remains incompletely understood; however, there is a clear link to various mutations, with a majority of patients having desmosomal abnormalities. It is thought that this loss of desmosomal integrity is key to the pathogenesis of ARVC. However, non-desmosomal mutations have also been seen to play a role in ARVC physiology [15]. Ventricular arrhythmias are a key feature of ARVC. Adipocyte deposition, which is seen with disease progression, is an essential point of interest. Despite this, the mechanisms behind adipogenesis remain an area requiring further research. More research must be conducted on these topics to reach a definitive conclusion about their mechanisms.

ARVC itself has a broad spectrum of clinical manifestations as patients can present asymptomatic, with presyncope/syncope, or even SCD. Many patients develop ventricular failure due to the structural changes leading to CHF symptoms [3]. Diagnosis is primarily performed through EKG and cardiac imaging studies, although a biopsy can also be performed for definitive diagnosis. There is also a role for genetic testing for asymptomatic relatives of ARVC patients. To ensure there is accurate and prompt diagnosis of ARVC, multiple guidelines have been developed.

Treatment for ARVC begins with conservative approaches and pharmaceutical intervention. However, given the risk of SCD, many patients eventually require ICD placement as their disease progresses. Ablation can also be pursued but is often not successful. In patients with refractory disease, cardiac transplant is a viable option [4].

This article provides a comprehensive overview of ARVC, including pathophysiology, genetics, clinical manifestations, and treatment. However, more research is needed to further our understanding of ARVC, particularly in disease genetics and pathology, which may help tailor further treatment options and diagnostic modalities.

## Figures and Tables

**Table 1:** A simplified list of genes implicated in arrhythmogenic right ventricular cardiomyopathy (ARVC).

Gene	Protein	Location
JUP	Plakoglobin	Desmosome
DSP	Desmoplakin	Desmosome
PKP2	Plakophilin 2	Desmosome
DSC2	Desmocollin 2	Desmosome
DSG2	Desmoglein 2	Desmosome
SNC5A	NAv1.5 sodium channel	Cardiac cell membrane
TTN	Titin protein	Sarcomere
CTNNA3	alpha-T catenin 3	Intercalated disk
CDH2	N-cadherin 2	Intercalated disk
TJP1	Tight junction protein 1	Intercalated disk
LMNA	Lamin A/B	Nuclear envelope
TMEM43	Luma	Nuclear envelope
PLN	Phospholamban	Sarcoplasmic reticulum
RYR2	Ryanodine 2 receptors	Sarcoplasmic reticulum
DES	Desmin	Intermediate filaments
TGFB3	Transforming growth factor beta 2	Cytokine

**Table 2:** A Comparison of the 2010 International Task Force ARVC Criteria and the 2024 European Task Force ARVC Criteria. ARVC: arrhythmogenic right ventricular cardiomyopathy. MRI: magnetic resonance imaging. RV: right ventricle. RBBB: right bundle branch block. SAEKG: signal-averaged electrocardiogram. LBBB: left bundle branch block. VT: ventricular tachycardia. NSVT: Non-sustained Ventricular Tachycardia. VF: ventricular fibrillation [51].

Category	2010 International Task Force ARVC Criteria	2024 European Task Force ARVC Criteria
Morpho-Functional Ventricular Abnormalities	Major Echo · Severe dilation and reduction of RV ejection fraction · Localized RV aneurysms · Severe dilation of RV only MRI · Regional RV dyskinesia, akinesia, or dyssynchronous RV contraction RV Angiography · Regional RV dyskinesia, akinesia, or aneurysm Minor Echo · Mild global RV dilation and/or reduced ejection fraction · Mild segmental dilation of RV · Regional RV hypokinesis MRI · Regional RV dyskinesia, akinesia, or dyssynchronous RV contraction	By Echocardiography, Cardiac MRI, or Angiography Major · Regional or Global akinesia, dyskinesia, or aneurysm with additional finding ○ Global RV dilation ○ Global RV systolic dysfunction Minor · Regional RV akinesia, dyskinesia, or aneurysm
Structural Alterations	Major · Residual myocytes <60% on morphometric analysis or <50% in estimated counts. Fibrous replacement of right ventricular wall without or without fatty tissue replacement Minor · Residual myocytes 60–75% on morphometric analysis or 50–65% in estimated counts. Fibrous replacement of right ventricular wall without or without fatty tissue replacement	Major · Fibrous replacement of myocardium in >1 sample with or without fatty replacement on EMB Minor · RV LGE in >1 RV region except for tricuspid valve on CMR
Repolarization Abnormalities	Major · Inverted T waves in Leads V1, V2, V3 in those >14 years old without RBBB Minor · Inverted T waves in leads V1 and V2 in patients >14 years old without RBBB. Or in leads V3-V6 · Inverted T waves in leads V1-V4 in presence of RBBB for those >14 years old	Major · Inverted T-waves in right precordial leads (V1, V2, V3) or beyond in individuals past puberty (age > 14 years) all in the absence of RBBB Minor · Inverted T-waves in V1 and V2 in males >14 years old all in the absence of RBBB · Inverted T-waves past V3 in individuals >14 years old with RBBB · Inverted T-waves past V3 in individuals <14 years old
Depolarization Abnormalities	Major · Epsilon waves in precordial leads Minor · Late potentials on SAEKG in >1 of 3 parameters in absence of QRS > 110 ms · Filtered QRS duration > 114 ms · Duration of terminal QRS < 40 uV but >38 ms · Root square mean voltage of terminal 40 ms is <20 uV · Terminal activation duration of QRS > 55 ms measured from the nadir of S wave to the end of QRS (R’ in V1, V2, V3) without RBBB	Minor · Epsilon Wave in right precordial leads (V1-V3) · Terminal activation of QRS is greater than >55 ms when measured from the nadir of S-wave to the end of QRS. All including the R’ wave in leads V1, V2, or V3, all in the absence of RBBB
Ventricular Arrhythmias	Major · NSVT or Sustained VT with LBBB morphology and superior axis · Negative or indeterminate complexes in inferior leads and positive complexes in aVL Minor · NSVT or Sustain VT with RV outflow configuration, LBBB with inferior axis (positive in inferior leads, and negative in aVL) or unknown axis · >500 ventricular extrasystoles per 24 h	Major · Frequent > 500 PVC with NSVT or sustained VT with LBBB morphology with non-inferior axis Minor · Frequent > 500 PVC with NSVT or sustained VT with LBBB morphology with inferior axis (RVOT pattern) · History of cardiac arrest due to VF or Sustained VT of unknown morphology
Familial History	Major · ARVC in 1st-degree relative · ARVC identified in autopsy · Identification of pathogenic gene associated with ARVC in suspected patient Minor · Identification of likely pathogenic gene in an individual being evaluated for ARVC · History of ARVC in 1st-degree relative but cannot confirm the diagnosis · Premature sudden death (<35 yrs) of 1st-degree relative with suspicious ARVC · ARVC confirmed in 2nd-degree relative	

## Data Availability

Not applicable.
